# A distribution-oriented approach to support landscape connectivity for ecologically distinct bird species

**DOI:** 10.1371/journal.pone.0194848

**Published:** 2018-04-11

**Authors:** José M. Herrera, Diogo Alagador, Pedro Salgueiro, António Mira

**Affiliations:** 1 CIBIO-InBIO/UE, Centro de Investigação em Biodiversidade e Recursos Genéticos—Universidade de Évora (CIBIO/InBIO-UE), Dom Augusto Eduardo Nunes 7, CP, Évora (Portugal); 2 Departamento de Ecología Integrativa, Estación Biológica de Doñana (EBD-CSIC), Avd. Americo Vespucio 26, CP, Seville (Spain); University of Porto, PORTUGAL

## Abstract

Managing landscape connectivity is a widely recognized overarching strategy for conserving biodiversity in human-impacted landscapes. However, planning the conservation and management of landscape connectivity of multiple and ecologically distinct species is still challenging. Here we provide a spatially-explicit framework which identifies and prioritizes connectivity conservation and restoration actions for species with distinct habitat affinities. Specifically, our study system comprised three groups of common bird species, forest-specialists, farmland-specialists, and generalists, populating a highly heterogeneous agricultural countryside in the southwestern Iberian Peninsula. We first performed a comprehensive analysis of the environmental variables underlying the distributional patterns of each bird species to reveal generalities in their guild-specific responses to landscape structure. Then, we identified sites which could be considered pivotal in maintaining current levels of landscape connectivity for the three bird guilds simultaneously, as well as the number and location of sites that need to be restored to maximize connectivity levels. Interestingly, we found that a small number of sites defined the shortest connectivity paths for the three bird guilds simultaneously, and were therefore considered key for conservation. Moreover, an even smaller number of sites were identified as critical to expand the landscape connectivity at maximum for the regional bird assemblage as a whole. Our spatially-explicit framework can provide valuable decision-making support to conservation practitioners aiming to identify key connectivity and restoration sites, a particularly urgent task in rapidly changing landscapes such as agroecosystems.

## Introduction

The ability of organisms to move through a landscape is a fundamental determinant of population persistence [1]. This is particularly true in human-impacted landscapes as successful movements may counteract the impacts of habitat loss and fragmentation by, for example, enabling organisms to forage over multiple habitat patches, rescuing populations from local extinction and promoting the colonization of new habitat patches [2–4]. Maintaining or increasing the extent to which the landscape facilitates the movements of organisms (landscape connectivity *sensu* [5]), is therefore widely recognized as an overarching strategy for conserving biodiversity in human-impacted landscapes [6]. However, determining landscape connectivity for multiple and ecologically distinct species still poses several theoretical and practical challenges, limiting the potential for conservation and management [7–9].

Inferring landscape connectivity requires a thorough knowledge of how organisms perceive a certain landscape and how they respond to changes in its structural properties [10,11]. Because obtaining real movement data, such as GPS telemetry data (e.g. [12]), is logistically and financially intensive, this information is commonly unavailable for multiple species in a landscape. Thus, landscape connectivity is most often inferred through the spatial distribution of species (e.g. [13–15]). The hypothesis underlying this approach is that the absence of a species in a given patch can be explained by the compositional and configurational attributes of the landscape, which make such a patch inaccessible or unsuitable for the species of concern (e.g. [16–18]). A burgeoning research literature suggests that spatial distribution patterns of species depend not only on the amount and spatial configuration of preferred land-cover types (e.g. natural forests for forest associated species), but also on the structure of the other landscape fractions (i.e. the landscape matrix) [19–21]. This is because the landscape matrix may offer suitable resources and environmental conditions for multiple species [22, 23]. However, most studies to date have focused principally on the characteristics of the presumed o estimated preferred land-cover type(s) rather than on the structural properties of the entire landscape mosaic. In this context, the development of a mosaic-based distribution-oriented approach to support landscape connectivity for multiple and ecologically distinct species continue to be a valuable contribution to conservation research [10].

Here, we performed an analysis of landscape connectivity for species with distinct habitat affinities with the aim of providing a spatially-explicit framework that prioritizes connectivity conservation and restoration actions. We focused on three groups of common bird species, forest-specialists, farmland-specialists, and generalists, populating a highly heterogeneous agricultural countryside in the southwestern Iberian Peninsula. We first performed a detailed analysis of the landscape-related mechanisms underlying the distributional patterns of each bird species to uncover generalities in their guild-specific response to landscape structure [24]. Then, using this information, we recreated a cost surface to represent landscape permeability to movement for the three individual bird guilds [25]. Through a connectivity optimization approach, our analysis first aimed to determine those sites that were critical in optimizing connectivity for each of the three species guilds, which might be considered important for conservation [26]. Secondly, we sought to determine the number and location of sites that would need to be restored to increase landscape connectivity [27]. To determine the number and identity of these sites, we quantified the critical number of restored cells above which connectivity metrics did not change. Those cells most frequently selected were considered key for restoration purposes.

## Methods and materials

### Study area and sampling framework

**Fig 1:**
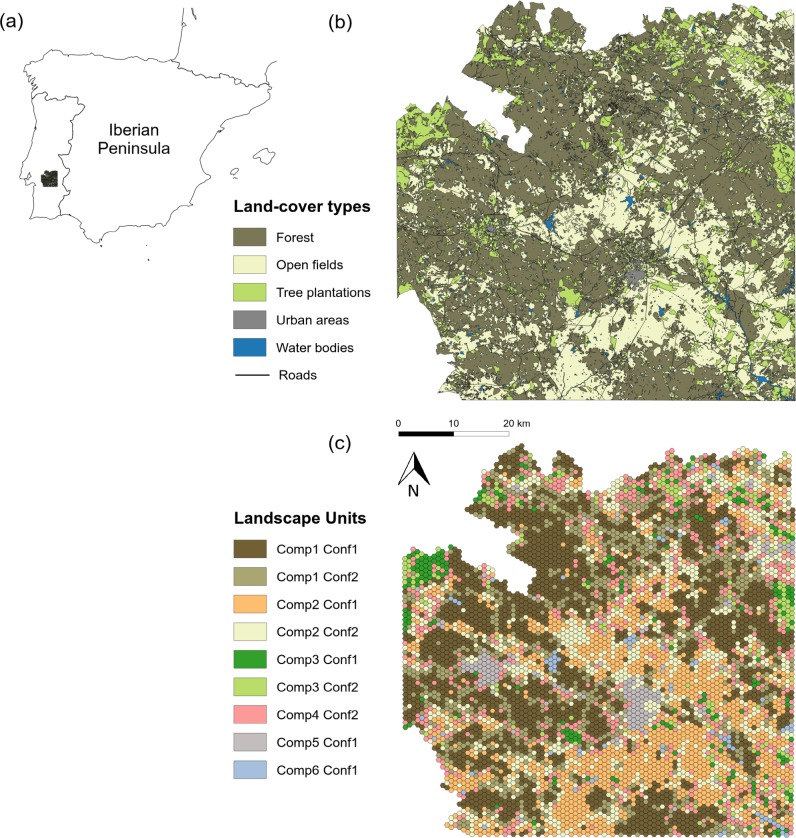
Landscape composition and configuration of the study region. Location of the study region (a). The different land-cover types and their spatial configuration across the study region (b) and the landscape units resulting from the integration of composition (Comp) and configuration (Conf) categories are depicted. This figure was produced by JMH using QGIS Development Team, 2015. QGIS Geographic Information System. Open Source Geospatial Foundation.

This work was carried out in the region of Alentejo in Southern Portugal (centroid: 16271.45, -113395.21; EPSG: 3763-ETRS89 / Portugal TM06), in a study area of *ca*. 426,000 hectares (Fig 1A; see also Fig 1 in [22]). The owners of land properties gave permission to conduct sampling surveys. The topography is flat, with altitude ranging between 100 and 450 m a.s.l. The climate is Mediterranean, typically warm and dry for a large part of the year, with summer temperatures reaching up 40 degrees Celsius and relatively mild but wetter winters. The landscape of the region mainly consists of savanna-like forests of cork (*Quercus suber* L.) and holm-oak (*Q*. *rotundifolia* L.) at varying densities, comprising the characteristic Portuguese *montado*, a High Nature Value Farming System according to the classification of the European Environmental Agency ([28], see also [29] for a detailed description of the system). The regional landscape also includes large open agricultural areas for cattle grazing and cereal farming as well as orchards of woody crops such as olive (*Olea europaea* L.) and vineyards (*Vitis* spp.), and timber plantations (mainly of *Pinus pinaster* L. and *Eucalyptus* spp.), all of which are interspersed with small human settlements, roads and wetlands (Fig 1B).

The study area was mapped using homogeneous gridding, which was applied to extract both landscape structural variables and species distribution data (see Landscape characterization). Rather than rectangular or square grids, we used hexagonal cells (henceforth referred to as ‘cells’) as they have been shown to be particularly suitable in spatially-complex landscapes [30]. We used a grid with a total area of about 65 ha composed of hexagons of 500-m radius, a cell size that has been demonstrated to be suitable for establishing bird–landscape relationships in multi-species approaches [31]. The entire study area comprised 6,767 cells.

### Landscape characterization

We characterized the landscape using land-cover data from CORINE Land-cover (CLCN5), the most detailed and highest resolution (1:25,000) database available for continental Portugal [32]. Because of the large number of land-cover classes included in CLCN5 (*n* > 200), we first integrated the land-cover types into six groups: (*i*) forests, including all types of natural woodlands such as *montado*), (*ii*) open agricultural areas, including extensive cereal farming and cleared forest areas used for cattle grazing, (*iii*) pine and eucalyptus plantations as well as and tree-like crops such as olive orchards and vineyards, (*iv*) urbanized areas, including urban and industrial areas, (*v*) transportation infrastructures such as roads and railroad tracks and (*vi*) water bodies, including reservoirs, rivers and watering holes.

The regional landscape was characterized using both composition and configuration metrics [22, 24]. Landscape composition was quantified based on the proportions of the six land-cover types detailed above within individual cells. Landscape configuration was estimated using the following variables: number of land-cover classes present in individual cells, Shannon diversity index for habitat types, mean number of patches, patch size, and the distance between patches of the different land-cover types. All landscape metrics were extracted by distinguishing between dominant and minority land-cover types to obtain independent landscape metrics. Dominant land-cover types were defined as those representing more than 25% of the cell area (i.e. ≥ 16.0 ha). Finally, composition and configuration variables were grouped to classify every cell as belonging to a given composition and configuration class using a multivariate clustering method, the *k*-means clustering algorithm [33]. We identified six compositional classes corresponding to cells dominated by: natural woodlands (Composition class 1), open agricultural lands (Composition class 2), tree plantations and/or woody crops (Composition class 3), no specific land-cover type, i.e. mixed cells (Composition class 4), urban areas (Composition class 5) or water bodies (Composition class 6).

Landscape configuration was also varied but fell into two distinct classes: homogeneous and heterogeneous cells. Homogeneous cells were characterized by large, scarce and close patches of dominant land-cover types (Configuration class 1). Heterogeneous cells were characterized by a high diversity of small and dispersed land-cover types (Configuration class 2). See Table 1 for a detailed description of each landscape composition and configuration class. Finally, each individual cell was characterized by combining both composition and configuration classes to form what is henceforth referred to as ‘landscape units’. Nine types of landscape units were therefore identified throughout the study region (Table 2).

**Table 1:** Mean (±SE) values of landscape variables used for characterizing landscape composition (a) and configuration (b) classes. Numbers in brackets represent minimum and maximum values. Dominant land-cover types are those which accounted for more than 25% of each 65 ha hexagonal cell (500-m radius) which were used to extract landscape structural variables and species distribution data.

(a)	Composition class	Composition description	Forest (%)	Open fields (%)	Plantations (%)	Urban (%)	Wetlands (%)			
	1	Strictly forest	82.4 ± 14.3 [43.3,100]	13.3 ± 0.2 [0,32.2]	2.1 ± 0.0 [0,28.1]	1.6 ± 0.0 [0,19.3]	0.9 ± 0.0 [0,19.7]			
	2	Strictly agriculture	17.8 ± 0.3 [0,32.2]	77.1 ± 0.3 [41.1,100]	2.8 ± 0.1 [0,29.5]	1.0 ± 0.0 [0,19.8]	1.1 ± 0.0 [0,19.5]			
	3	Plantations	12.1 ± 0.6 [0,43.5]	11.2 ± 0.6 [0,44.9]	74.7 ± 0.7 [52.3,100]	1.2 ± 0.1 [0,18.3]	0.6 ± 0.0 [0,14.0]			
	4	Mixed	34.3 ± 0.7 [0,71.0]	31.4 ± 0.6 [0,68.0]	31.1 ± 0.4 [7.4,53.1]	2.0 ± 0.1 [0,19.5]	0.9 ± 0.0 [0,19.5]			
	5	Urban	12.9 ± 1.9 [0,50.1]	30.1 ± 2.1 [0,42.1]	1501 ± 1.1 [0,55.2]	52.2 ± 2.1 [30.2,100]	0.49 ± 0.1 [0,6.5]			
	6	Wetlands	30.1 ± 2.6 [0,50.1]	28.2 ± 2.4 [0,68.0]	5.2 ± 1.2 [0,49.2]	3.5 ± 1.6 [0,4.5]	31.6 ± 1.1 [36.5,100]			
					Dominant land-cover types			Minority land-cover types		
(b)	Configuration class	Configuration description	Shannon equitability	Number of land-cover types (n)	Number of patches (n)	Patch size (ha)	Patch distance (m)	Number of patches (n)	Patch size (ha)	Patch distance (m)
	1	Homogeneous cells	0.4 ± 0.0 [0,1.4]	2.7 ± 0.0 [1,2]	1.1 ± 0.0 [1,4]	31.5 ± 0.3 [0,64]	42.4 ± 1.0 [0,406.3]	0.6 ± 0.0 [0, 4]	5.2 ± 0.1 [0,32.4]	37.3 ± 0.8 [0,365.7]
	2	Heterogeneous cells	0.8 ± 0.0 [0.1.5]	3.8 ± 0.0 [2,5]	2.7 ± 0.0 [1,10.5]	10.2 ± 0.1 [0,61.4]	211.2 ± 2.7 [0,816.7]	1.6 ± 0.0 [0,7]	6.7 ± 0.1 [0,32.1]	124.2 ± 2.3 [0,963.2]

**Table 2:** Total number (N total) of cells belonging to each type of landscape unit and number of cells where bird censuses was carried out (Nsampled). Expected frequency distribution, calculated as N sampled / N total × 100, and real frequency distribution for forest-specialist, farmland-specialist, and generalist species are also reported. The suitability of each landscape unit for each bird guild was determined by comparing the real frequency distribution to the expected frequency distribution. Any landscape unit with a real frequency between 0–5% above the expected frequency distribution for a guild was considered as passage, while those landscape units showing real values 5% above or below expected values were identified as suitable and unsuitable, respectively.

Composition description (class)	Configuration description (class)	N total	N sampled	Expected frequency distribution (%)	Real frequency distribution (%)			Suitability		
					Forest	Farmland	Generalist	Forest	Farmland	Generalist
Strictly forest (1)	Homogeneous (1)	2128	57	35.2	62.1	22.5	45.1	suitable	unsuitable	suitable
	Heterogeneous (2)	1069	23	14.2	16.1	17.1	15.9	passage	passage	passage
Strictly agriculture (2)	Homogeneous (1)	1257	36	22.2	6.0	33.8	13.4	unsuitable	suitable	unsuitable
	Heterogeneous (2)	971	15	9.2	5.7	13.4	9.2	unsuitable	suitable	passage
Plantations (3)	Homogeneous (1)	206	12	7.5	2.6	4.7	4.3	unsuitable	unsuitable	unsuitable
	Heterogeneous (2)	199	9	5.5	4.9	4.8	5.6	unsuitable	unsuitable	passage
Mixed (4)	Homogeneous (1)	0	0	–	–	–	–	–	–	–
	Heterogeneous (2)	677	10	1.4	2.6	3.7	6.5	passage	passage	passage
Urban (5)^a^	Homogeneous (1)	191	0	–	–	–	–	unsuitable	unsuitable	unsuitable
Wetlands (6)^a^	Homogeneous (1)	69	0	–	–	–	–	unsuitable	unsuitable	unsuitable
Total		6777	162							

^a^Cells allocated to these landscape units were arbitrarily considered as being unsuitable for all the bird guilds studied in this work.

### Bird sampling and landscape suitability

Birds were sampled during the 2013 breeding season (April–May) in a total of 162 cells. Field surveys did not involve endangered or protected species. Sampling surveys were reviewed and approved by the Institute for Nature and Forest Conservation (ICNF; Portuguese government). To adequately sample birds across the complete range of structural complexity of the study area, we selected cells corresponding to each type of landscape unit while accounting for their representativeness throughout the regional landscape. Since landscape patterns are commonly locally-aggregated, a robust sampling design was essential to reach a comprehensive understanding of the influence of landscape structure on species distribution [34]. Sampling effort across landscape units was distributed on the basis of their relative frequency. Thus, the higher frequency of appearance a given landscape unit, the higher the number of sampling sites selected for bird surveys (Table 2). Individual cells selected for sampling purposes within each landscape unit were randomly selected. Cells from all Composition and Configuration classes were selected for bird sampling except those corresponding to Composition class 5, which were excluded from the analysis due to their low representativeness (2.8% and 1.0% respectively; Table 2). We alternated between the type of landscape units across sampled plots to avoid differences in the composition of the bird assemblage between landscape units as a result of variations in sampling time. To ensure spatial representativeness in the distribution of species, we discarded bird species occurring in fewer than 10 sampled cells from statistical models to increase the discriminatory power between suitable and unsuitable sites [35]. For a complete list of species and number of sampled cells in which they occurred, see S1 Table.

Sampling consisted of 10-minute point counts where the presence of every bird visually observed or heard within a radius of 100 m (~3.14 ha) was recorded. Any sign of breeding activity (e.g. singing males, territorial behavior, nest construction or provisioning) was also recorded. Birds were sampled once at the center of each selected cell in sessions that began just after sunrise and concluded no later than 10:00 a.m. to avoid the central hours of the day when bird activity is at its minimum in the study region [36]. Based on our objectives, we reduced survey effort per site while increasing the number of sites surveyed to gain statistical power and improve the representativeness of the data for the area studied [37, 38]. To obtain comparable data between sampled cells, bird counts were performed by the same observer (PS), controlling for observer bias. Surveys in cloudy (above 50% cloud cover), rainy and windy (wind greater than 10mph) were avoided and were all performed in similarly favorable weather conditions [38]. Sites were never visited for sampling purposes in the presence of aerial predators. Flyovers were excluded from analysis. Weather conditions (rain, cloud cover, temperature, wind speed) were monitored to obtain comparable data between sampled plots. Each species was classified into a guild of habitat specialization following the classification of the Pan-European Common Bird Indicator [39]; species were defined as either forest-specialist, farmland-specialist, or generalist (S1 Table).

Finally, every cell was classified as either suitable, passage or unsuitable for each guild by comparing the observed frequency distribution of species across landscape units to the expected values derived from the relative frequency of each landscape unit in the study region [24]. A landscape unit where the frequency of a given bird guild showed little or no deviation from expected values (i.e. ± 5%) were considered as having neither positive nor negative impact on such species (i.e. passage areas). In contrast, those landscape units in which the frequency of a bird guild were above or below 5% relative to the expected values were considered as being suitable and unsuitable, respectively (Table 2).

### Connectivity analysis

To perform the connectivity analysis, we used the publicly available software *MulTyLink* [9], which uses principles and algorithms from graph theory to determine cost-efficient connectivity linkages between environmentally suitable areas using a spatially explicit approach [40]. *MulTyLink* is particularly suitable for our purposes because it is specifically designed to retrieve efficient linkages for species or group of species with distinct ecological requirements. Thus, when selecting areas for a given species or group of species in a graph, *MulTyLink* considers the possibility of using these selected areas for other species (or group of species), thus reducing linkage cost (e.g. in terms of length or area) [9]. Here, the cost associated with passage and unsuitable landscape units for each bird species group was set as one (*c*_*i*_ = 1), and that for suitable landscape units as zero (*c*_*i*_ = 0). It should be mentioned that, with no detailed information on the differential impacts of different passage areas for each species group, we chose to define costs homogeneously for those areas. Moreover, the costs defined for unsuitable areas assume that they may be passage areas for some species groups, and therefore, the same assumption applies. In this way solutions are insensible to the particular value of costs. In this way, the optimization process to determine cost-efficient connectivity linkages between environmentally suitable areas, thus operated on the basis of minimizing the amount of passage areas (i.e. number of cells selected), while fixing (at no cost) the suitable habitats for the three species groups. It is worth noting that because *MulTyLink* was developed to operate using a network of square cells, our hexagonal cells were transformed into square cells (see S1 Fig for a detailed description of the adjustment process).

First, we conducted the connectivity analysis to determine the cells critical to maintaining current landscape connectivity, henceforth referred as key conservation cells. To perform this analysis, we obtained corridors for each individual guild using the *Grasp* (Greedy Randomized Adaptive Search Procedure) algorithm from *MulTyLink* [28, 41]. This algorithm was performed 100 times, retrieving the best connectivity linkage, defined as having the minimum cost, from 100 independent stochastic runs. We thus obtained 100 distinct solutions, each providing cost-efficient linkages between guild-specific suitable cells (Table 2). Next we determined the number of times each individual cell was selected during the 100 runs, and considered all those cells selected 100 of 100 times to be cells key for conservations.

To ascertain the ecological significance of these key conservation cells, we first ran *MulTyLink* 100 times over a simulated landscape where these cells were degraded into unsuitable habitats for all three bird guilds (e.g. tree plantations; Table 2). In parallel, we simulated 100 landscapes in which cell degradation was randomly allocated throughout the landscape, using the same number of degraded cells and key conservation cells. Then we compared the number of connected cells as a surrogate of the total connected area as well as the number and size of connected clusters for each bird guild as a surrogate of the degree of connectivity between suitable landscape units.

The second approach to our connectivity analysis consisted of a spatially-explicit prioritization exercise aimed to determine those cells that, if restored, would maximize landscape connectivity for the three individual guilds while ensuring the shortest movements for the whole bird assemblage. Thus, we simulated the impact on connectivity metrics of converting, for example, homogeneous tree plantations to heterogeneous forests or mixed habitats (Table 2). All the restored cells that were identified for connectivity linkages in all 100 runs were considered key restoration cells. To ascertain the ecological significance of this restoration option, we compared connectivity metrics using this approach to those resulting from the restoration of random locations (*n*_*r*_) within the landscape. These comparisons were made by starting with *n*_*r*_ equal to the number of key restoration cells and then adding or subtracting three cells at a time.

## Results

### Bird community

Overall, the presence of 68 bird species (S1 Table) were recorded. In total, 39 species (57.3%) were documented in at least 10 sampled cells and were included in the subsequent suitability and connectivity analysis (S1 Table). Of these, 10 species (25.6%) were considered as forest-specialists, 17 (43.5%) as farmland-specialists and 12 (30.9%) as generalists (S1 Table). The most abundant bird species (n = 367, 11.5%), also recorded in the highest number of cells (n = 129, 7.5%), was the corn bunting (*Emberiza calandra*; S1 Table). Other particularly abundant species were the Spanish sparrow *Passer hispaniolensis* (*n* = 276;8.6%), the common chaffinch *Fringilla coelebs* (*n* = 276;8.6%) and the Eurasian blue tit (*n* = 196;6.1%) (S1 Table). Both *Cyanistes caeruleus* (*n* = 106;6.1%) and *Fringilla coelebs* (*n =* 104;6.0%), as well as the short-toed tree-creeper *Certhia brachydactyla* (*n =* 79;4.5%), were also among the species occurring in a higher number of cells (S1 Table).

### Landscape suitability

**Fig 2:**
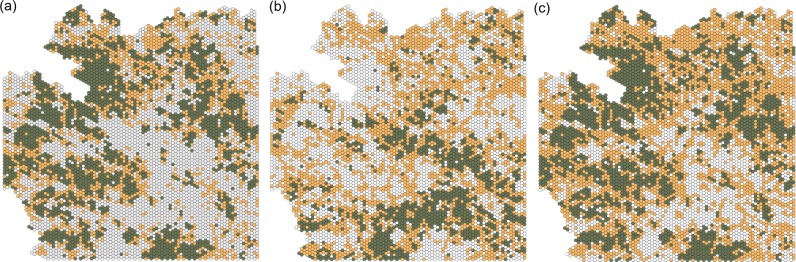
Maps of landscape suitability. Maps depicting the landscape suitability for (a) forest-specialists, (b) farmland-specialists and (c) generalist species. Different colors represent suitable (dark green), passage (light brown) and unsuitable (white) cells for each bird species group.

The proportion of cells classified as suitable throughout the study region was similar among bird guilds (31.4%, 37.2 and 31.4% for forest specialists, farmland specialists, and generalist species, respectively) (Table 2). The proportion of cells classified as passage, however, was higher for generalist species (48.4%) compared to that for forest (25.8%) and farmland specialists (25.8%). The proportion of cells classified as unsuitable was higher for forest specialists (42.7%), but similar to those estimated for farmland specialists (41.2%). Unsuitable cells were comparatively lower for generalist species (25.5%) (Table 2). Both suitable and unsuitable cells exhibited a clustered spatial pattern in all bird guilds, whereas passage cells exhibited a more dispersed spatial pattern (Fig 2).

The approach we used to determine landscape suitability for different bird species was able to identify biologically meaningful relationships between land-cover and bird guilds. Homogeneously forested cells (Composition class 1; Configuration class 1), for example, were identified as suitable landscape units for forest-specialist species, but not for farmland specialists (Table 2). Only strictly cropland cells (Composition class 2) were indeed identified as suitable landscape units for farmland species, irrespective of their spatial configuration (Table 2). Homogeneous cropland cells (Composition class 2; Configuration class 1) were unsuitable for both forest specialist and generalist bird species. However, heterogeneous cropland cells (Composition class 2; Configuration class 2) were identified as passage landscape units for generalist species (Table 2). Heterogeneous forested cells (Composition class 1; Configuration class 2), were identified as passage landscape units for all three guilds as were mixed cells (Composition class 4) (Table 2). Cells comprising homogeneous plantations were consistently found to be unsuitable landscape units for all three bird guilds, while heterogeneous plantation cells were identified as passage landscape units only for generalist species (Table 2).

### Landscape connectivity

Total connected area as estimated by the number of connected cells was 1.5 times higher for generalist species than for farmland- or forest-specialist species (Table 3A). The degree of connectivity between suitable landscape units, as estimated by the number and mean size of connected-patch clusters, was highest for forest specialists, followed by farmland-specialists and then by generalist species (Table 3A).

**Table 3:** Connectivity metrics, mean number of connected cells as well as the number and mean size of connected clusters, for the three scenarios investigated in the present study: baseline (a), degradation (b) and restoration (c) scenarios.

	a)	Baseline			b)	Degradation			c)	Restoration		
			Clusters				Clusters				Clusters	
		Connected cells	Number	Size		Connected cells	Number	Size		Connected cells	Number	Size
Forest-specialists		247.80 ± 0.62	33	5.15		208.75 ± 0.62	40	4.25		290.90 ± 0.61	19	8.95
Farmland-specialist		276.02 ± 0.65	21	7.23		233.85 ± 0.66	34	4.47		284.20 ± 0.44	16	9.50
Generalist species		344.15 ± 0.70	6	28.33		335.75 ± 0.83	8	21.25		341.50 ± 0.80	4	42.50
All		476.05 ± 0.44	60	8.20		465.85 ± 0.44	82	6.00		477.85 ± 0.41	39	12.62

**Fig 3:**
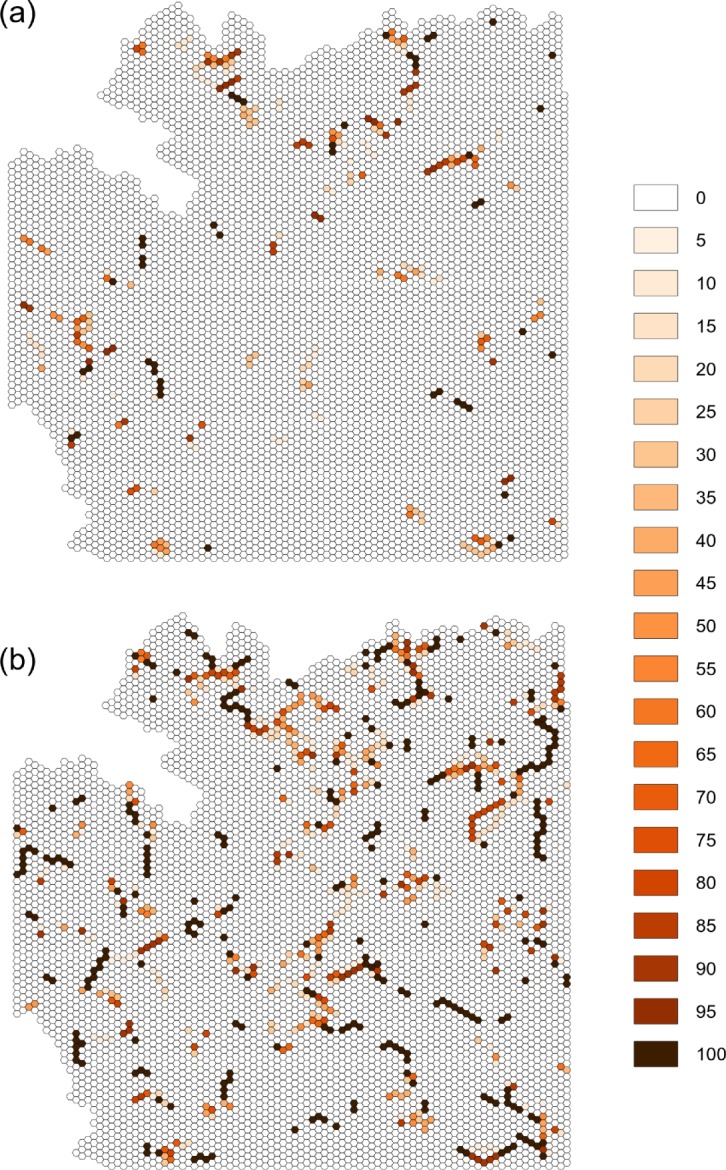
Spatial distribution and frequency selection of cells comprising corridors for the three guilds. The spatial distribution and frequency selection in the baseline landscape scenario (a) and after applying the restoration approach (b). The restoration approach consisted of restoring an increasing number of cells into suitable landscape units for the three bird guilds in a simulation process (*n* = 15; see Fig 4). Key conservation areas are those cells consistently selected (*n* = 100). The color legend with shades of orange/brown shows the number of times a cell was selected in the randomization process.

A total of 46 cells (2,990 ha) were selected in all 100 model replicates of movement linkage for each of the three bird guilds simultaneously, and were thus considered to be key conservation cells (Fig 3A). They were mainly composed of heterogeneous forests (76.1%; Composition class 1; Configuration class 2) and by mixed cells (23.9%; Composition class 4). The ecological significance of these key conservation cells was revealed after degrading them into unsuitable landscape units. Thus, the degradation of these cells reduced the total connected area and, more importantly, increased the degree of fragmentation of connected clusters in all bird guilds (Table 3; Fig 3).

**Fig 4:**
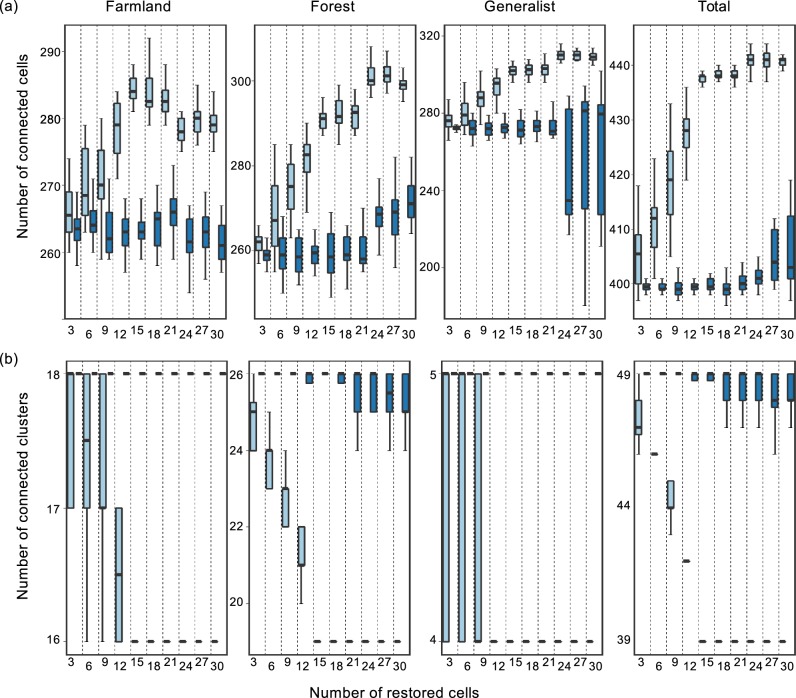
Variation of landscape connectivity metrics as a function of number of cells restored to suitable landscape units. Upper panels (a) show the response in total number of connected cells and lower panels (b) show the number of connected clusters. Boxplots represent variation in values over 100 runs for each set of restored cells. Horizontal lines represent medians, the bottom and top box edges are the 1_st_ and 3_rd_ quartile, respectively, and whisker lines represent 1.5 times the inter-quartile range. In each scenario, the left-most (light blue) boxes represent scenarios in which unsuitable landscape units for the three bird guilds simultaneously were converted into suitable or passage landscape units. The right-most (dark blue) boxes represent scenarios where cells for restoration were chosen randomly from across the study area, irrespective of guild-specific landscape suitability. Note the different scales of the *y*-axes in each case.

Both total connected area and number of clusters reached an asymptote when a total of 15 cells (975 ha) were restored from unsuitable to passage landscape units (Fig 4). Each of these cells consistently selected were therefore considered to be key restoration cells (Fig 3B). This restoration threshold was similar when accounting for each bird guild individually and for all three guilds together (Fig 4). Restoring these cells had a significant impact on landscape connectivity, particularly on the number and size of connected clusters (Table 3C; Fig 3). Interestingly, in the case of generalist bird species, the total connected area decreased under the restoration scenario even though a higher number of suitable habitat patches were linked together (Fig 4).

## Discussion

We describe a restoration-prioritization methodology that integrates both spatial-graphs and landscape suitability models, able to evaluate the impact of degrading and/or restoring key connectivity areas for multiple and ecologically distinct species. In doing so, we first investigated the landscape-related mechanisms underlying the spatial patterning of species distribution to infer guild-based landscape suitability for bird species. This landscape suitability was then used as a measure of resistance to movement. By using a spatially explicit approach, we finally identified priority sites as well as both the number and spatial location of sites that should be restored to significantly increase landscape connectivity for the regional bird community as a whole. Our approach provides valuable support to conservation practitioners in the decision-making process to identify key connectivity and restoration zones, a particularly challenging task in rapidly changing landscapes such as agroecosystems [42, 43].

Most research aiming to infer landscape connectivity from resistance values computed by landscape characteristics have assumed that the availability of preferred land-cover type plays an essential (and often exclusive) role in shaping the spatial distribution patterns of species [44, 45]. Nevertheless, because most landscapes, particularly human-modified landscapes, are often mosaics of various land-cover types that may offer a number of different suitable resources and environmental conditions [22], such an approach to landscape suitability analysis might be too simplistic and theoretically inadequate [46]. Landscapes are increasingly recognized continuous, multi-dimensional gradients of environmental suitability rather than discrete units of suitable versus non-suitable habitat [47]. To determine the influence of landscape structure on the distribution of species, we used a landscape-centered approach (*sensu* [46]) which uses spatial units of land-cover mosaics cells as replicates [20]. Because it is built independent of species-specific habitat requirements, this landscape-centered approach allowed us to determine landscape suitability for multiple and ecologically distinct bird species [22].

As expected, we found that homogeneously forested sites were suitable for forest-specialist bird species, but unsuitable for farmland-specialists. Interestingly, however, heterogeneous strictly forest cells were identified as passage habitats not only for forest specialists but also for farmland-specialists. This might be explained by the fact that heterogeneous strictly forest areas in our study region are typically savanna-like forests with varying densities of trees (the characteristic Portuguese *montado* [29]), resulting in both forest- and farmland-specialists perceiving these sites as areas of inferior quality for their use as territory but suitable as areas of passage [48,49]. In this context, our results also suggest that the impact of increasing heterogeneity is stronger for forest-specialist species than for farmland-specialist species, as only homogeneous strictly forested sites were found to be suitable for forest-specialists, while both homogeneous and heterogeneous strictly agricultural areas were suitable for farmland-specialists. We also found that homogeneous tree plantations were unsuitable for all three guilds of bird species studied here, supporting existing knowledge demonstrating that tree plantations are poor habitats for native birds [50–52]. Indeed, this type of habitat was only used as passage areas by generalist birds when tree plantations were heterogeneously distributed. Overall, our findings support the widely accepted conclusion that both properties of landscape structure, landscape composition and configuration, are critical in delineating species distribution patterns [22, 46].

The new approach we present here provides a spatially-explicit framework which identifies and prioritizes connectivity conservation and restoration actions for species with distinct habitat affinities. This is possible because (1) it incorporates landscape suitability and connectivity requirements for each single species or a group of species, (2) it enables the identification of priority sites for the maintenance of current landscape connectivity (i.e. key conservation sites), and (3) it identifies sites whose restoration would permit maximum landscape connectivity. Most distribution-oriented approaches to support landscape connectivity for multiple species rely on the identification of focal or umbrella species [14, 48, 49]. However, this poses a challenge because sites suitable for dispersal for one species might be impermeable to others [13–15, 48, 49]. By accommodating connectivity requirements for multiple species, our approach has the potential to provide valuable contributions to research for biodiversity conservation and management. Our approach was able to recognize priority sites for conservation and management at the landscape level. In particular, some sites were highlighted as conservation priorities, as their structural properties and spatial location allow the movements of the three bird guilds. Moreover, our approach was able to identify those areas for which restoration from unsuitable to passage habitats for the three bird guilds facilitates a reduction in landscape fragmentation [27]. Interestingly, only a small fraction of the landscape (*n* = 15 cells) was identified as a priority for restoration to minimize fragmentation for the three bird guilds studied. From a management perspective, habitat restoration in these areas would permit maximum landscape connectivity at minimum cost.

## Supporting information

S1 Table Bird species recorded and their relative abundance across landscape units. (ODT)

S2 Table Raw environmental and bird data. (XLS)

S1 Fig Changes performed in *MulTyLink* for using hexagonal cells. (DOC)
