# Supplementary material for: A distribution-oriented approach to support landscape connectivity for ecologically distinct bird species
Source: PLoS One. 2018 Apr 11;13(4):e0194848. doi: 10.1371/journal.pone.0194848 (PMC5895004; doi:10.1371/journal.pone.0194848)

**S1 Figure** *MulTyLink* was originally developed to take analyses over a regular grid of square cells. However, with some geo-referenced data manipulation *MulTyLink* can be adjusted to retrieve results for a grid of hexagonal cells. This is implemented through the tuning of the dispersal parameter to be defined for each species group (i.e., forest specialists, farmland specialists and generalists; see the main text for details). This parameter defines a neighborhood set for each focal hexagonal cell, and therefore indicates for where a species can move (if not an unsuitable barrier cell). Therefore, we transformed the original geo-referential space into a simpler one where the *y*-distance between the centroids of lateral neighboring hexagons was settled to a step-unit, *u* while their *x-*distance to was settled to 2*u*.Within this transformed grid the distance (*D*) defining the von Neumann neighborhood of each hexagonal cell (light grey cells) is settled in the range 2.23 < *D* < 3.61.


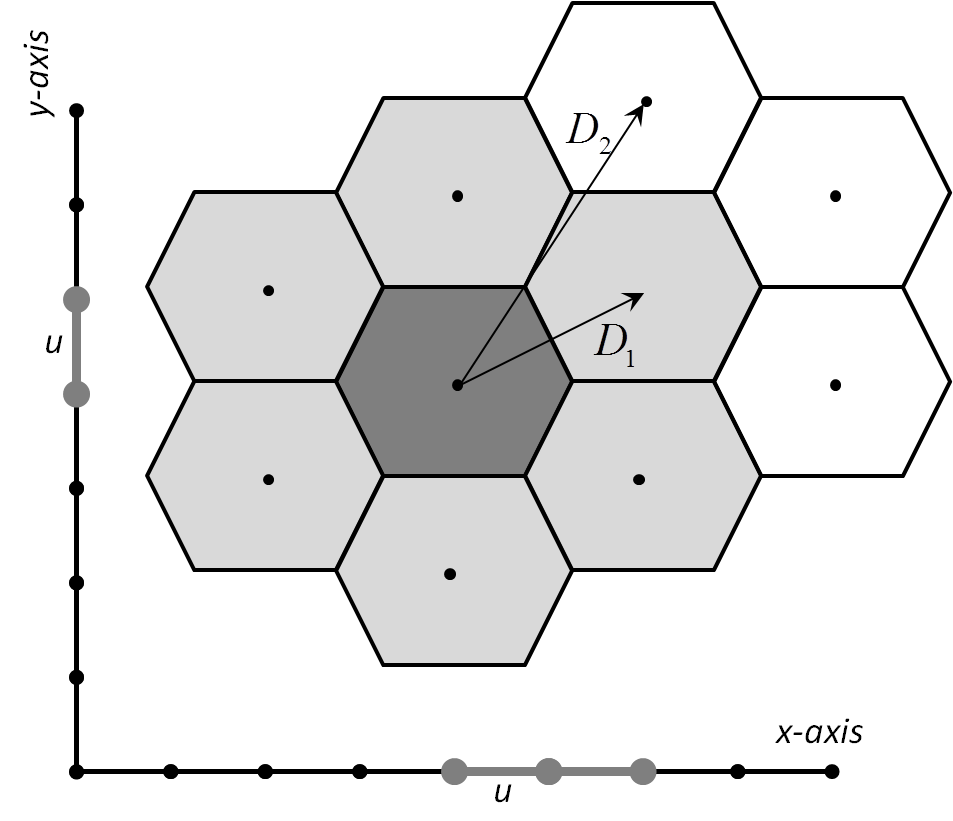

Supplement: S1 Fig — (DOC) [file pone.0194848.s003.doc]
