# Supplementary material for: A distribution-oriented approach to support landscape connectivity for ecologically distinct bird species
Source: PLoS One. 2018 Apr 11;13(4):e0194848. doi: 10.1371/journal.pone.0194848 (PMC5895004; doi:10.1371/journal.pone.0194848)
Supplement: Supplementary file 4 [file pone.0194848.s001.odt]

S1 Table. List of the bird species recorded in this study as well as the number of hexagonal cells in which they were sampled (Nc) and their total abundance (Ta). The species-specific habitat association of those species present in more than 10 cells (i.e., those marked with the † symbol) and therefore those included in subsequent connectivity analysis is also reported (n = 41). See the main text for details.

|  |  |  |  |  |
| --- | --- | --- | --- | --- |
| Scientific name | Common name | Group | Nc | Ta |
| Alcedo atthis L. 1758 | Common Kingfisher |  | 1 | 1 |
| Alectoris rufa L. 1758† | Red-legged Partridge | Farmland | 16 | 21 |
| Apus pallidus Shelley 1870 | Pallid Swift |  | 1 | 1 |
| Ardea cinerea L. 1758 | Grey Heron |  | 3 | 4 |
| Bubulcus ibis L. 1758† | Cattle Egret | Farmland | 12 | 40 |
| Burhinus oedicnemus L. 1758 | Stone-curlew |  | 1 | 1 |
| Calandrella brachydactyla Leisler 1814 | Greater Short-toed Lark |  | 1 | 1 |
| Carduelis cannabina L. 1758† | Common Linnet | Farmland | 24 | 44 |
| Carduelis carduelis L. 1758† | European Goldfinch | Farmland | 68 | 118 |
| Carduelis chloris L. 1758† | European Greenfinch | Generalist | 39 | 49 |
| Cecropis daurica L. 1771 | Red-rumped Swallow |  | 4 | 11 |
| Certhia brachydactyla Brehm 1820† | Short-toed Treecreeper | Forest | 79 | 145 |
| Cisticola juncidis Rafinesque 1810† | Zitting Cisticola | Farmland | 55 | 74 |
| Clamator glandarius L. 1758 | Great Spotted Cuckoo |  | 4 | 5 |
| Coccothraustes coccothraustes L. 1758 | Hawfinch |  | 4 | 7 |
| Columba palumbus L. 1758† | Common Woodpigeon | Forest | 16 | 17 |
| Corvus corone L. 1758† | Carrion Crow | Farmland | 21 | 24 |
| Corvus monedula L. 1758 | Eurasian Jackdaw |  | 1 | 5 |
| Coturnix coturnix L. 1758† | Common Quail | Farmland | 41 | 52 |
| Cuculus canorus L. 1758† | Common Cuckoo | Generalist | 31 | 35 |
| Cyanistes caeruleus L. 1758† | Common Blue Tit | Forest | 106 | 196 |
| Cyanopica cooki Bonaparte 1850 | Iberian Azure-winged Magpie |  | 2 | 10 |
| Delichon urbicum L. 1758 | Northern House Martin |  | 1 | 13 |
| Dendrocopos major L. 1758† | Great Spotted Woodpecker | Forest | 10 | 10 |
| Dendrocopos minor L. 1758 | Lesser Spotted Woodpecker |  | 4 | 5 |
| Egretta garzetta L. 1766 | Little Egret |  | 1 | 1 |
| Emberiza calandra L. 1758† | Corn Bunting | Farmland | 129 | 367 |
| Emberiza cirlus L. 1766 | Cirl Bunting |  | 6 | 6 |
| Erithacus rubecula L. 1758 | European Robin |  | 1 | 2 |
| Erythropygia galactotes Temminck 1820 | Rufous Scrub-robin |  | 1 | 1 |
| Estrilda astrild L. 1758 | Common Waxbill |  | 1 | 2 |
| Fringilla coelebs L. 1758† | Common Chaffinch | Forest | 104 | 276 |
| Galerida cristata L. 1758 | Crested Lark |  | 5 | 9 |
| Galerida theklae Brehm 1858† | Thekla Lark | Farmland | 59 | 120 |
| Garrulus glandarius L. 1758 | Eurasian Jay |  | 4 | 5 |
| Hippolais polyglotta Vieillot 1817† | Melodious Warbler | Farmland | 16 | 17 |
| Hirundo rustica L. 1758† | Barn Swallow | Farmland | 15 | 31 |
| Jynx torquilla L. 1758 | Eurasian Wryneck |  | 1 | 1 |
| Lanius meridionalis Temminck 1820† | Southern Grey Shrike | Farmland | 21 | 23 |
| Lanius senator L. 1758† | Woodchat Shrike | Farmland | 24 | 29 |
| Lophophanes cristatus L. 1758† | European Crested Tit | Forest | 12 | 15 |
| Lullula arborea L. 1758† | Woodlark | Farmland | 71 | 102 |
| Luscinia megarhynchos Brehm 1831† | Common Nightingale | Generalist | 61 | 81 |
| Melanocorypha calandra L. 1766 | Calandra Lark |  | 6 | 15 |
| Merops apiaster L. 1758† | European Bee-eater | Generalist | 30 | 73 |
| Oriolus oriolus L. 1758 | Eurasian Golden Oriole |  | 7 | 7 |
| Parus major L. 1758 | Great Tit |  | 1 | 1 |
| Passer domesticus L. 1758† | House Sparrow | Generalist | 23 | 55 |
| Passer hispaniolensis Temminck 1820† | Spanish Sparrow | Farmland | 27 | 276 |
| Passer montanus L. 1758† | Eurasian Tree Sparrow | Forest | 47 | 54 |
| Petronia petronia L. 1766 | Common Rock-sparrow |  | 7 | 8 |
| Phoenicurus phoenicurus L. 1758 | Common Redstart |  | 3 | 3 |
| Pica pica L. 1758† | Common Magpie | Generalist | 13 | 20 |
| Riparia riparia L. 1758 | Collared Sand Martin |  | 1 | 1 |
| Saxicola torquatus L. 1766† | Common Stonechat | Farmland | 42 | 55 |
| Serinus serinus L. 1766† | European Serin | Forest | 55 | 82 |
| Sitta europaea L. 1758† | Eurasian Nuthatch | Forest | 47 | 69 |
| Streptopelia decaocto Frivaldszky 1838† | Eurasian Collared-dove | Generalist | 44 | 52 |
| Sturnus unicolor Temminck 1820† | Spotless Starling | Generalist | 62 | 133 |
| Sylvia atricapilla L. 1758† | Blackcap | Generalist | 14 | 21 |
| Sylvia hortensis Gmelin 1789 | Orphean Warbler |  | 2 | 3 |
| Sylvia melanocephala Gmelin 1789† | Sardinian Warbler | Generalist | 71 | 103 |
| Tetrax tetrax L. 1758† | Little Bustard | Farmland | 10 | 16 |
| Troglodytes troglodytes L. 1758† | Northern Wren | Forest | 19 | 23 |
| Turdus merula L. 1758† | Common Blackbird | Generalist | 62 | 78 |
| Turdus viscivorus L. 1758 | Mistle Thrush |  | 1 | 1 |
| Upupa epops L. 1758† | Hoopoe | Generalist | 51 | 53 |
|  |  |  |  |  |
